# Subtype-Specific Ligand Binding and Activation Gating in Homomeric and Heteromeric P2X Receptors

**DOI:** 10.3390/biom14080942

**Published:** 2024-08-02

**Authors:** Xenia Brünings, Ralf Schmauder, Ralf Mrowka, Klaus Benndorf, Christian Sattler

**Affiliations:** 1 Institute of Physiology II, Jena University Hospital, Friedrich Schiller University Jena, 07743 Jena, Germany; xenia.bruenings@med.uni-jena.de (X.B.); ralf.schmauder@med.uni-jena.de (R.S.); 2 Experimentelle Nephrologie, KIM III, Universitätsklinikum Jena, Nonnenplan 4, 07743 Jena, Germany; ralf.mrowka@med.uni-jena.de; 3 ThIMEDOP—Thüringer Innovationszentrum für Medizintechnik-Lösungen, Nonnenplan 4, Universitätsklinikum Jena, 07743 Jena, Germany

**Keywords:** P2X receptors, fluorescent ATP, patch-clamp, ligand binding, membrane proteins, Mg^2+^ modulation

## Abstract

P2X receptors are ATP-activated, non-specific cation channels involved in sensory signalling, inflammation, and certain forms of pain. Investigations of agonist binding and activation are essential for comprehending the fundamental mechanisms of receptor function. This encompasses the ligand recognition by the receptor, conformational changes following binding, and subsequent cellular signalling. The ATP-induced activation of P2X receptors is further influenced by the concentration of Mg^2+^ that forms a complex with ATP. To explore these intricate mechanisms, two new fluorescently labelled ATP derivatives have become commercially available: 2-[DY-547P1]-AHT-ATP (fATP) and 2-[DY-547P1]-AHT-α,βMe-ATP (α,βMe-fATP). We demonstrate a subtype-specific pattern of ligand potency and efficacy on human P2X2, P2X3, and P2X2/3 receptors with distinct relations between binding and gating. Given the high in vivo concentrations of Mg^2+^, the complex formed by Mg^2+^ and ATP emerges as an adequate ligand for P2X receptors. Utilising fluorescent ligands, we observed a Mg^2+^-dependent reduction in P2X2 receptor activation, while binding remained surprisingly robust. In contrast, P2X3 receptors initially exhibited decreased activation at high Mg^2+^ concentrations, concomitant with increased binding, while the P2X2/3 heteromer showed a hybrid effect. Hence, our new fluorescent ATP derivatives are powerful tools for further unravelling the mechanism underlying ligand binding and activation gating in P2X receptors.

## 1. Introduction

**Figure 1:**
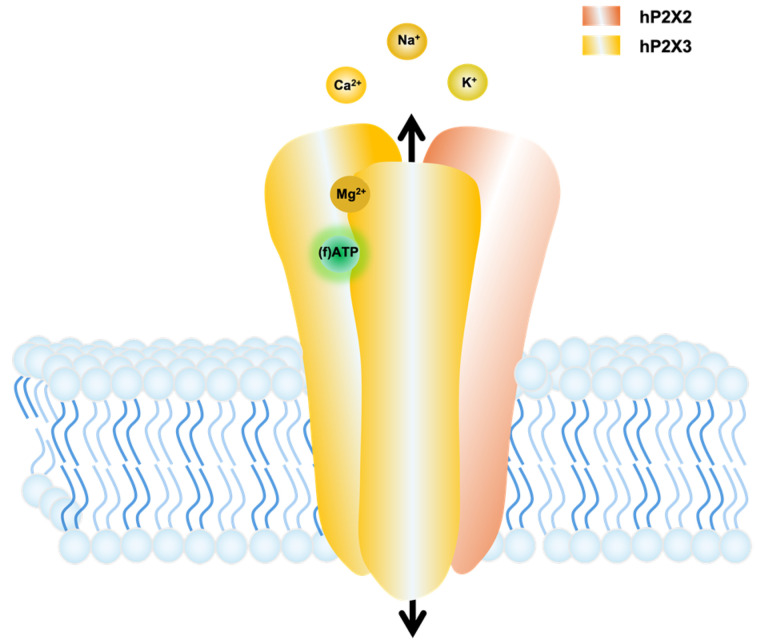
Cartoon illustrating the interaction between a heterotrimeric P2X2/3 receptor with the ligands (f)ATP and Mg^2+^. The extracellular domain contains three principally different sites between the subunit interfaces for (f)ATP binding. Here, we show only one binding site as an example. ATP binding results in the ion flux. Mg^2+^ ions can bind in complex with ATP to the orthosteric binding site and, additionally, to an allosteric binding site at the P2X receptor, resulting in activation and modulation of its function, respectively.

Ionotropic purinergic receptors, also known as P2X receptors, are ion channels that become active upon ATP binding to extracellular binding sites, allowing cations to pass through their pore. Mammals exhibit seven subunit isoforms (P2X1 to P2X7), forming either homotrimeric or heterotrimeric membrane proteins (Figure 1) [1,2]. P2X receptors are expressed in various tissues and play relevant roles in multiple physiological and pathophysiological processes such as pain, inflammation, taste, and synaptic transmission [3]. Understanding the specific roles of the individual P2X receptors is desirable for developing specific treatments for diverse diseases. All P2X receptors share a common trait: an ectodomain forms an extracellular loop, along with two transmembrane segments, TM1 and TM2, carrying the intracellular N- and C-termini, respectively [1,4].

Six of the seven cloned P2X isoforms can efficiently form functional homotrimeric ion channels and exhibit different gating and pharmacological properties. One relevant difference between them is their apparent affinity (*EC*_50_) for the ligand ATP, which ranges from low micromolar to millimolar concentrations [5]. Additionally, the speed of desensitisation varies among the seven isoforms. Homomeric P2X1 and P2X3 channels desensitise quickly in tens of milliseconds, and their recovery from desensitisation is extremely slow and agonist-dependent [6]. This slow recovery constrains the possible frequency of channel activation, potentially serving as a safeguard against sensory hypersensitivity in a living cell. Previous research has indicated that the release of an agonist from the desensitised receptor contributes to the recovery from desensitisation at the P2X1 and P2X3 receptors [7,8,9,10]. However, the precise molecular mechanisms underlying these distinctive gating processes are still awaiting elucidation [11]. P2X2, P2X4, P2X5, and P2X7 take much longer to desensitise, ranging from many seconds to minutes, or they do not desensitise at all. Heteromeric P2X2/3 receptors have a slow desensitisation pattern akin to P2X2. The unique pharmacological and electrophysiological properties of P2X receptor subtypes can be used to discriminate between the P2X receptors, since the potency and efficacy of the ATP analogues vary among the receptor subtypes. P2X3 can be activated by αβmethylene-ATP (αβMe-ATP), an ATP analogue with a significantly higher affinity and efficacy for activating P2X3 compared to P2X2. αβMe-ATP is also a full agonist at P2X2/3 receptors, but with a slightly reduced potency compared to P2X3 receptors. These unique characteristics offer a method to differentiate P2X2/3 heteromeric channels from P2X2 or P2X3 homomeric channels [7,12,13]. Because P2X3 homotrimers and P2X2/3 heterotrimers have very similar pharmacological properties, it seems that the P2X2 subunit controls the basic biophysical properties in the heterotrimer, while the P2X3 subunit defines its pharmacological properties [1,14,15].

Regarding the ATP, it is well established that only a small fraction of ionised ATP^4−^ is present in vivo. ATP is predominantly found in complexes with divalent ions, especially as Mg^2+^ATP and Ca^2+^ATP [16,17]. While free ATP acts as an agonist in controlled laboratory settings, P2X receptors predominantly respond to Ca^2+^ATP and Mg^2+^ATP in physiological contexts. This preference arises from the fact that ATP released from cells at micromolar concentrations primarily forms complexes with Ca^2+^ and Mg^2+^, both abundant in millimolar concentrations in the extracellular space [11,16,18]. This observation has implications for the understanding of the physiological role of ATP within biological systems. Several studies have investigated the effect of divalent ions on the kinetics and gating behaviour of P2X receptors [16,19,20]. The effects of Mg^2+^ATP and ATP^4−^ vary across different subtypes of P2X receptors. P2X2 receptors can be activated by free ATP, whereas Mg^2+^ATP shows minimal efficacy in promoting opening. Conversely, free ATP and Mg^2+^ATP effectively open the rapidly desensitising P2X3 subtype. Another notable difference between these subtypes is how Mg^2+^ regulates P2X3 receptors through a unique allosteric mechanism [16]. Heteromeric P2X2/3 channels in sensory neurons display a mixed phenotype, featuring strong activation by Mg^2+^ATP and minimal regulation by Mg^2+^ [16]. In this context, the recovery of P2X3 receptors from desensitisation is a gradual process: temporary elevations in extracellular Mg^2+^ significantly and reversibly reduced ATP-evoked currents that had not yet recovered from desensitisation. A Ca^2+^-free solution has a similar effect as Mg^2+^. These findings suggest that the P2X3 receptors on sensory neurons can be inhibited by high concentrations of Mg^2+^ or a lack of Ca^2+^, representing a negative feedback mechanism to limit ATP-mediated nociception [21].

Located at the interfaces of the three subunits, the three ATP binding sites initiate a rapid and substantial conformational change throughout the receptor upon ligand binding, ultimately resulting in pore opening. Various approaches, including site-directed mutagenesis, electrophysiological recordings, molecular modelling, and X-ray crystallography, have elucidated the mechanism by which ATP binding is linked to gating [22,23,24,25,26]. Besides ATP, recent studies also used fluorescently labelled ATP derivatives (fATP) to decipher the molecular mechanisms in P2X receptors [27,28]. To use this approach, a prerequisite is that the fluorescent ligand acts as a potent full agonist and exhibits a brightness suitable for optical recording. We previously synthesised and functionally characterised the fluorescent ATP derivative 2-[Dy-547P1]-AET-ATP, which exhibited a sufficiently intense fluorescence when bound to the channels [27]. This allowed us to relate ligand binding to activation gating for P2X2 receptors. In the case of the alternative fluorescent ATP derivative, BODYPY-TR ATP, the dye was linked to the ribose moiety [29]. This derivative was employed to investigate the agonist-induced movement of the ATP-binding jaw [29]. Monitoring both ligand binding and activation gating enabled the identification of amino acids outside the binding pocket that influence access to it [29]. A third fluorescent ATP derivative, Alexa Fluor^®^ 647 adenosine 5’triphosphate (Alexa-647-ATP), Thermo Fisher Scientific (Dreieich, Germany), was used to study the desensitisation of P2X1 receptors and their recovery process [28].

Herein, we used two newly synthesised fluorescent ATP derivatives based on our previously reported 2-[DY-547P1]-AET-ATP, first with an elongated linker between the ATP and the dye 2-[DY-547P1]-AHT-ATP, and second, the dye 2-[DY-547P1]-AHT- α,βMe-ATP (Section 3.1) with a methylene group in the phosphate chain to target a subtype-specific activation. We tested these ATP derivatives on human P2X2, P2X3, and P2X2/3 receptors using the patch-clamp technique (Section 3.2) and optical binding experiments in cell cultures (Section 3.3). In the case that both ATP and fATP are referenced, we specify this in the following by ‘(f)ATP’. Additionally, we studied the effect of Mg^2+^ on binding and gating (Section 3.4). Our work demonstrates a subtype-specific ligand–protein interaction for P2X receptors, as well as its modulation by Mg^2+^.

## 2. Materials and Methods

### 2.1. Chemicals

The chemicals for buffers were from either Roth (Karlsruhe, Germany) or Sigma Aldrich (Taufkirchen, Germany). The restriction enzymes and buffers for subcloning were purchased from Thermo Fisher Scientific (Dreieich, Germany). Primers were purchased from Eurofins (Ebersberg, Germany). Polymerases were purchased from HighQu (Kraichtal, Germany). Poly-L-lysine and apyrase were purchased from Sigma Aldrich. ATP was purchased from Sigma Aldrich and α,βMe-ATP was purchased from Trocis. The ATP derivatives 2-[DY-547P1]-AHT-ATP (fATP) and 2-[DY-547P1]-AHT- α,βMe-ATP (α,βMe-fATP) were purchased from Biolog (Bremen, Germany)**.** The reference dye DY647 was purchased from Dyomics GmbH (Jena, Germany).

### 2.2. Molecular Biology and Cell Culture

The human P2X2 splice isoform B (hP2X2) and P2X3 (hP2X3) were amplified from cDNA (BD Biosc. 4030043) and subcloned with respective restriction sites in pcDNA5/FRT/TO. For the co-expression of hP2X2 and hP2X3 subunits, a plasmid with two expression cassettes containing the tetracycline-inducible CMV promoter, the hP2X gene, and the BGH termination sequence was created using the PCR technique and unique restriction sites. All plasmids were verified via restriction analysis and partial sequencing.

Human P2X receptors were expressed from HEK293 cell lines containing an inducible promoter system (Flp-In-T-REx 293, Invitrogen, Waltham, MA, USA). The used cell lines were: hP2X2 B (RRID: AC line CVCL_D6U1), hP2X3 (RRID: AC line CVCL_D6U3), and hP2X2/3 (RRID: AC line CVCL_D6U2). The cells were cultured in MEM supplemented with non-essential amino acids (Gibco, Waltham, MA, USA), 10% FCS, and antibiotics, according to the manufacturer’s instructions. For stable cell lines, Flp-In-T-REx 293 cells were transfected using the calcium phosphate method with a mixture of plasmids (0.3 µg pcDNA5/FRT/TO P2X gene and 1.5 µg pOG44) and selected for hygromycin resistance. The cells were seeded on glass coverslips for electrophysiological measurements and used 24–48 h after tetracycline induction (1 µg/µL).

### 2.3. Electrophysiology

Membrane currents were recorded with a standard patch-clamp technique [30] in the whole-cell configuration. The patch pipettes were pulled from borosilicate glass (ID 1.0 mm, OD 2.0 mm; VITROCOM, Mountain Lakes, NJ, USA) using a micropipette puller (P-97, Sutter Instrument, Novato, CA, USA). The pipettes were filled with intracellular solution containing (mM) 142 NaCl, 5 BAPTA, 5 EGTA, and 10 HEPES at pH 7.4. The pipette resistance was from 2.0 to 6.0 MΩ. The bath solution contained (mM) 142 NaCl, 10 EGTA, 10 HEPES, and 10 glucose at pH 7.4. To increase the speed of recovery from the hP2X3 receptors, we additionally used a bath solution containing (mM) 142 NaCl, 10 HEPES, 10 glucose, 2 CaCl_2_, and 1 MgCl_2_. The cells were lifted for recording from the chamber bottom by the patch pipette and positioned in front of the outlet of the application pipette.

Solutions switches were carried out with the application pipette that had three barrels (inner diameter ~600 µm, Warner Instruments) controlled by a step motor (SF-77B, Warner Instruments). The speed of the laminar solution flow out of the barrels was estimated to be 2–5 cm/s. One barrel contained a control solution and another was connected to a solution selector (Vici Valco Instruments), which allowed us to apply different test solutions by exchanging the solution during the interval (60 sec for P2X2 and P2X2/3 receptors, 180 s for P2X3 receptors) of applying the control solution. Cells expressing hP2X3 were washed with 3 Units/mL apyrase for 30 min to remove ATP. To ensure the comparability of the data points between each application, we rinsed hP2X3 with the following solution for 3 min after each ATP application (mM): 142 NaCl, 10 HEPES, 10 glucose, 2 CaCl_2_, and 1 MgCl_2_ at pH 7.4. After sufficient recovery, we switched to the bath solution for 10 s and then to the solution of interest. The speed of the switch around a whole cell was estimated to be below 10 ms by switching between different salt solutions.

The saturation of activation was determined with ATP at 100 µM. The currents were recorded with a HEKA EPC 10 amplifier in combination with the patchmaster software. The sampling rate was 10 kHz and the recordings were on-line filtered at 2.9 kHz using a four-pole Bessel filter. The currents were recorded at a constant holding potential of −50 mV and the series resistance was compensated with the patchmaster software up to 80%.

### 2.4. Confocal Microscopy

**Figure 2:**
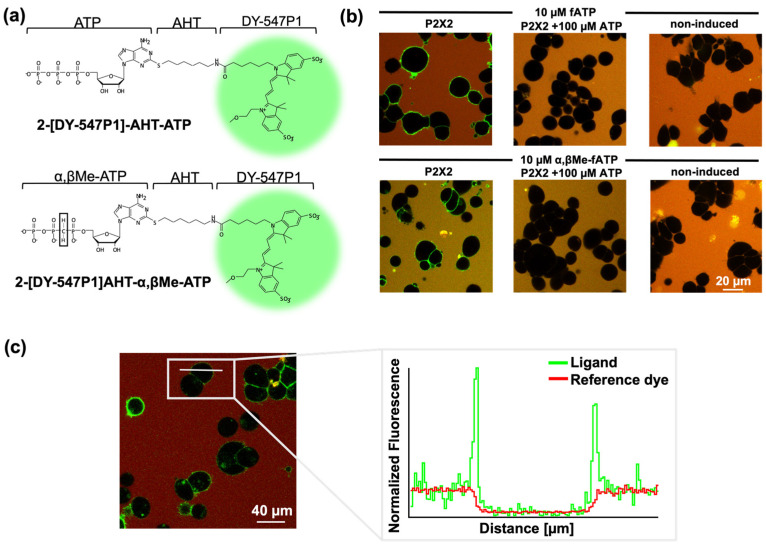
Confocal microscopy was used to characterise the binding of fluorescently tagged ligands to HEK 293 cells stably expressing human P2X2 receptors. (**a**) Structure of fATP and α,βMe-fATP. The dye DY547P1 is attached to the 2-position of the purine ring through an aminohexylthio-linker. (**b**) Representative confocal images for quantification of fATP and α,βMe-fATP binding, including approximately 200 stably transfected cells/mm^2^. Specific binding is proven by the lack of signal in non-induced cells and in cells expressing P2X2 receptors in the presence of 100 µM ATP. (**c**) To measure the binding of the fluorescently labelled ATP to P2X2 receptors in HEK cells, an automated analysis was conducted.

For the detection of ligand binding, the cells were incubated with the fluorescent ATP derivatives in chambered coverslips or 96-well plates with cover-slip bottoms. The surface was coated with 0.1 mg/mL of poly-L-lysine for 12 h and washed twice with PBS before seeding the cells at 22 cells/mm^2^ density. The cells were suspended 24 h after induction and allowed to settle for two hours before being utilised for the measurements. The solutions were freshly prepared before each experiment. The bulk solution was counter-stained with the red fluorescence dye Dy647. Each confocal voxel covering the cell membrane contained both the signal from the bound ligand and the signal from ligands in the bulk solution. This additional signal, e.g., the fraction of the bulk solution inside a voxel, varied from pixel to pixel and can hardly be described theoretically. Therefore, a scaled difference image between the images of the labelled ligand and reference fluorophore shows the pure binding signal (illustrated in Figure 2c).

Because the difference in image noise contributed within both the bulk solution and the cells, the cell–bulk interfaces were selected using a Sobel operator [31] and appropriate filters/thresholds on the reference fluorophore signal. Note that only cell–bulk boundaries and not cell–cell boundaries were included in this approach. The method was described in detail previously [27].

### 2.5. Quantification and Statistical Analysis

The concentration–activation relationships from whole-cell measurements in the HEK293 cells were obtained from the maximum currents during ATP application. These current amplitudes were normalised with respect to the current at saturating ATP (100 µM) and the resulting data points were fitted

*I*/*Imax*_ATP_ = *Amp*/(1 + (*EC*_50_/[X])^h^) (1)

with the Origin 2019^®^ software using a non-linear curve fitting routine. *I* is the actual current amplitude and *Imax*_ATP_ is the maximum current amplitude at saturating ATP. The amplitude *Amp* is the efficacy of the respective ligands. *EC*_50_ is the ligand concentration generating the half-maximum current and h is the respective Hill coefficient. [X] is the actual concentration of ATP to be tested.

The concentration–binding relationships were normalised to their maximum relative fluorescence intensities at a saturating fATP or α,βMe-fATP concentration.

Time-dependent activation and deactivation were described by fitting the respective time courses with a single exponential function yielding *t_on_* for activation and *t_off_* for deactivation, respectively. The speed of activating hP2X3 receptors was too fast to be described with a single exponential function because of the overlapping sigmodal time course due to the solution exchange. Therefore, we determined the rise time between 10% and 90% activation and calculated a *t_on_* = *ln*(9) × *t*_10/90_ for an assumed exponential function to compare the data with the other recordings.

The concentrations of Mg^2+^ATP and ATP^4−^ in solutions with varying concentrations of MgCl_2_ and ATP were calculated using Maxchelator 2019, as described by Bers et al. [32].

Errors are given as mean ± SEM. Statistical significance was assessed by using the Student’s *t*-test for unpaired data or the Mann–Whitney-U test when appropriate. A one-way ANOVA with Turkey correction was used to determine statistical significance between multiple experimental groups. The numerical and statistical analysis of the data was carried out by using the Origin.Lab 2019 and Fitmaster 2019 software. Differences with a *p*-value less than 0.05 are considered as significant.

## 3. Results

### 3.1. Novel Fluorescent ATP Derivatives and Visualisation of Ligand Binding

Measuring the binding of ligands to receptors is challenging. Receptors are complex and can exist in multiple conformations or undergo modulation, making it difficult to measure their binding properties accurately. Presently, it is not entirely clear how the signal of ligand binding spreads and opens the channel pore. Numerous questions remain unanswered, such as the nature of subunit cooperativity and the propagation of ligand binding. Here, we used fluorescently labelled ATP (fATP) and α,βMe-ATP (α,βMe-fATP) derivatives, with the dye DY-547P1 coupled to the purine ring via an amino-hexyl-thio linker, to investigate the binding and activation gating of P2X receptors (Figure 2a). Ligand binding was measured in stably transfected HEK293 cells expressing P2X receptors, attached to the glass bottom of the experimental chamber (Figure 2b). Quantifying ligand binding by automated fluorescence methods remains demanding at low expression levels and high ligand concentrations when the background signal becomes significant. To address this issue, we used the red fluorescence dye Dy647 at a concentration of 1 µM [27,33,34]. This allowed us to create a scaled difference image between the labelled ligand and reference dye, resulting in a pure binding signal (Figure 2c). With this approach, we could reliably measure fATP binding up to a concentration of 17.32 μM. Figure 2b displays typical images utilised for quantifying the binding signals. The elimination of the binding signal with fATP and α,βMe-fATP in the presence of an excess of ATP validated the specific binding to the orthosteric binding site.

### 3.2. Subtype-Specific Ligand Response in P2X Receptor Proteins

**Figure 3:**
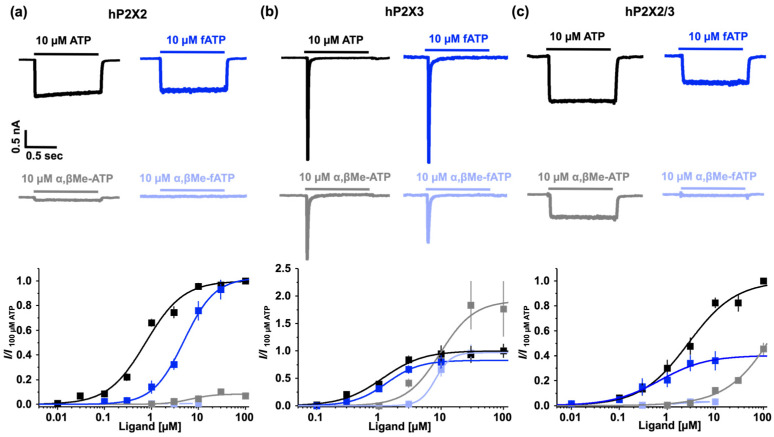
Activation of P2X receptors by different ligands. Representative current recordings of different ligands and concentrations-activation relationships from human P2X2 receptors (**a**), P2X3 receptors (**b**), and P2X2/3 receptors (**c**). The maximum amplitude of the current signals was normalised with respect to the maximum current amplitude at 100 µM ATP. Fluorescent ATP derivatives were normalised to 100 µM ATP due to high costs. Means of n = 5–17 cells (±SEM) were fitted with Equation (1) to obtain values for *EC*_50_ and H (Table 1). Records from HEK293 cells stably expressing the respective receptors in the whole-cell configuration at −50 mV.

It is well established that different P2X receptor subtypes possess distinct biophysical and pharmacological properties. We investigated two novel fluorescent ATP derivatives on three P2X receptors. To evaluate the efficacy and potency of fATP and α,βMe-fATP as agonists, we compared the current responses at identical saturating ATP concentrations (Figure 3). The measurements were performed in a divalent-cation-free bath solution containing 10 mM EGTA. This strategy allowed us previously to analyse high apparent affinities for rat P2X2 and P2X7 receptors [27,35]. Each fluorescent ligand showed unique pharmacological characteristics.

Fluorescent ATP acts as a full agonist at P2X2 receptors (*EC*_50_ = 4.6 µM) and exhibits a similar potency but slightly reduced efficacy at human P2X3 receptors. The potency of fATP at the P2X2 receptors was reduced (~0.2-fold), while α,βMe-ATP had an *EC*_50_ of ~6.8 µM (Table 1). As far as we know, these are the highest reported apparent affinities for ATP and α,βMe-ATP [36]. However, when coupled with a fluorophore, α,βMe-ATP did not evoke a current response. In the P2X3 receptors, α,βMe-ATP showed an unexpectedly high efficacy compared to ATP (~1.9-fold), with comparable *EC*_50_ values for the fluorescent and non-fluorescent ligands (ATP: 1.1 µM, fATP: 1.3 µM, α,βMe-ATP: 9.6 µM, α,βMe-fATP: 8.1 µM).

The established *EC*_50_ value of 2.7 µM ATP for the human P2X2/3 receptor matches prior findings [37]. However, significantly higher concentrations of the agonist α,βMe-ATP would have been necessary to determine an *EC*_50_, which we did not explore due to the costs. Although fATP is a partial agonist for the P2X2/3 receptor, it exhibits a notable potency increase of approximately 4-fold. While α,βMe-fATP did not show large amplitudes, it was a potent ligand with an *EC*_50_ value of 1.5 µM. Therefore, the addition of a fluorophore reduced the efficacy at P2X2/3 receptors, but increased potency. Furthermore, coupling ATP or α,βMe-ATP with a fluorophore decreased the potency compared to ATP and α,βMe-ATP at the P2X2 receptors, but did not affect the potency at the P2X3 receptors. The methylene group reduced the ligand potency across all receptors and decreased the efficacy at the P2X2 receptors, abolishing it entirely for the fluorescent ligand at the P2X2 receptors. Overall, among the receptors, ATP exhibited a higher apparent affinity than α,βMe-ATP.

### 3.3. P2X Receptor Subtypes Exhibit Unique Ligand Binding Characteristics

**Figure 4:**
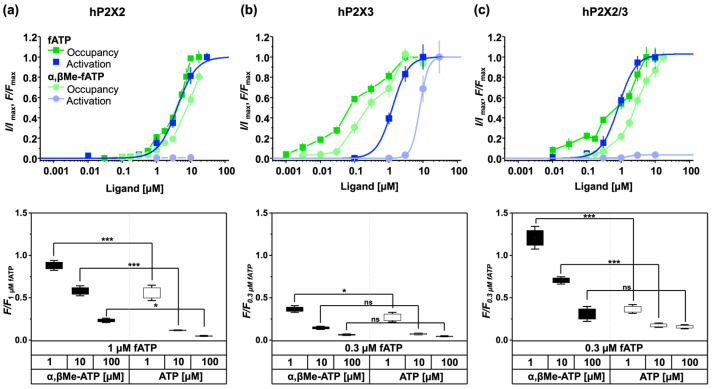
Concentration–binding relationships and concentration–activation relationships with fATP and α,βMe-fATP for human P2X2 receptors (**a**), P2X3 receptors (**b**), and P2X2/3 (**c**) receptors. The figure shows the concentration–binding and concentration–activation relationships and below competition assays for the corresponding receptor, which demonstrates the ability of fATP (1 µM for hP2X2 and 0.3 µM for hP2X3 and hP2X2/3) to compete with both ATP and α,βMe-ATP. The reference signal of 1 µM fATP (hP2X2) and 0.3 µM fATP (hP2X3 and hP2X2/3) without competing with other ligands was used for normalisation. Each receptor exhibits unique binding affinities, with α,βMe-ATP being able to differentiate between hP2X3 and hP2X2 or hP2X2/3. The data points, which indicate a binding signal, are derived from 15–40 images and are presented as mean ± SEM. The concentration–activation and concentration–binding relationships are normalised to their respective maximal values, except for the small or negligible current amplitudes with α,βMe-fATP. * *p* < 0.05 and *** *p* < 0.001. ns—non significant.

All fluorescent ATP derivatives can report the degree of binding at the ATP binding pocket of all receptors. The concentration–binding relationships show multiple components (Figure 4). Generally, the binding data could not be described by a single or a double Hill function. Here, the data points were connected by lines.

**Figure 5:**
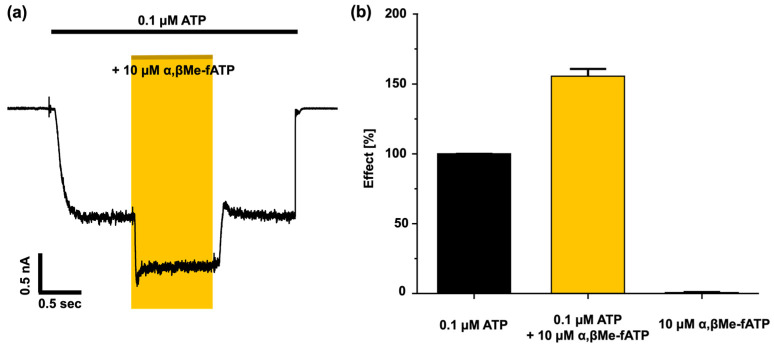
The current response to ATP and α,βMe-fATP at human P2X2 receptors. (**a**) The co-application of α,βMe-fATP (10 µM) with ATP (0.1 µM) elicits a notable increase in the current response. (**b**) Comparison of the current responses of ATP, α,βMe-ATP, and their co-application with 0.1 µM ATP.

Both fATP and α,βMe-fATP demonstrated a robust binding affinity to human P2X2 receptors, but α,βMe-fATP failed to reach a saturating concentration. The significant binding observed at the orthosteric binding site was unexpected given the low activation potency of α,βMe-ATP and the absence of activation by α,βMe-fATP. However, both α,βMe-fATP and α,βMe-ATP exhibited a strong binding affinity at the binding site, as indicated by the underlying data (Figure 4a). Therefore, the binding of α,βMe-fATP can report the degree of binding at closed P2X2 receptors. This raised the question of whether α,βMe-fATP exerted any unknown effects. To address this question, we co-applied 0.1 µM ATP and 10 µM α,βMe-fATP (Figure 5). α,βMe-fATP alone exhibited no discernible effect on the P2X2 receptors. However, co-applied with ATP, it demonstrated an additive, i.e., synergistic impact. The activation levels were elevated by approximately 55%. Therefore, the P2X2 receptors become responsive to α,βMe-fATP when partially pre-activated by 0.1 µM ATP. At the P2X3 receptors, the ligand exhibited binding to the channel at very low concentrations, but it did not effectively activate the receptor. Hence, in P2X3 receptors, the binding of fluorescent ATP derivatives reveals a notable leftward shift compared to the concentration–activation relationship.

fATP showed binding at the P2X2/3 receptors without relevant activity at low concentrations. By rescaling the concentration–activation relationship for fATP to the value at the maximal concentration, the two curves intersect at approximately 0.5 µM, indicating that, at higher concentrations, activation saturates before binding (Figure 4c). The ligand acts as a partial agonist, binding to the receptor with a high affinity, but only partially activating it.

### 3.4. Modulating P2X Receptor Channel Signalling by Mg^2+^

Investigations of P2X receptors with divalent ions are of great significance, as these ions are present in the human body in the millimolar range, predominantly bound to ATP, and they can play important regulatory roles. Understanding the role of divalent ions is crucial for gaining insights into essential aspects of the nervous system [16]. Lit et al. investigated the active states of ATP and observed differences in the effects of Mg^2+^ATP and ATP^4−^ across various P2X receptor subtypes. While P2X2 receptors can be activated by free ATP, Mg^2+^ATP facilitates opening with a low efficacy. Conversely, free ATP and Mg^2+^ATP robustly induce opening in both P2X3 and heteromeric P2X2/3 subtypes.

**Figure 6:**
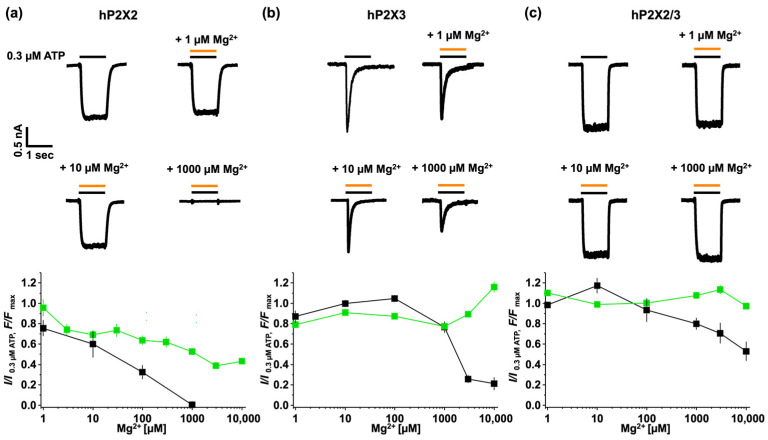
Binding and activation of Mg^2+^ATP. Mg^2+^ATP can bind to P2X2, P2X3, and P2X2/3 and activates P2X3 and P2X2/3, but not P2X2. Each panel displays representative current traces with 0.3 µM ATP (black line) and 0.3 µM ATP containing Mg^2+^ (orange line) for hP2X2 (**a**), hP2X3 (**b**), and hP2X2/3 (**c**). Below, the relationship between binding and activation is depicted for each respective subtype as a function of the Mg^2+^ concentration. The data points show the binding of fATP (green line) obtained from 15–40 images (mean ± SEM). The ATP response (black line) is shown as mean ± SEM from 5–11 cells.

**Figure 7:**
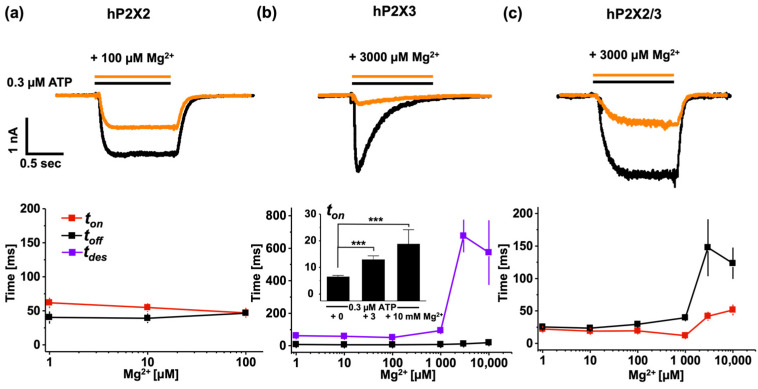
Time courses of activation (*t_on_*), deactivation (*t_off_*), and desensitisation (*t_des_*) of Mg^2+^ modulated ATP-induced currents of P2X receptors. Mg^2+^ changed time constants only at high Mg^2+^ levels in human P2X3 receptors (**b**) and human P2X2/3 receptors (**c**), but not in human P2X2 receptors (**a**). *** *p* < 0.001.

It was hypothesised that Mg^2+^ATP binds to the P2X2 receptor with a significantly lower apparent affinity than ATP alone, resulting in less efficient channel opening [16]. To address this question, we used fATP to examine the effect of Mg^2+^ binding and activation on the P2X2 receptor (Figure 6). The results showed that, for P2X2 receptors, there was a Mg^2+^ concentration-dependent reduction in activation. As the concentration of Mg^2+^ increased, the current decreased. No activation was observed at 1000 µM Mg^2+^. While binding gradually decreased with an increasing Mg^2+^ concentration, significant binding was still detectable, even after the application of 10 mM Mg^2+^. These results indicated that, while ~40 percent of Mg^2+^fATP remained bound, it did not activate the channel (Figure 6a). Interestingly, the time constants for activation and deactivation remain unchanged, despite the drastic effects on binding and activation (Figure 7a).

In contrast, the P2X3 receptor showed that activation remained relatively unaffected up to 100 µM Mg^2+^ and only showed reduced activation at higher Mg^2+^ concentrations. Although activation started to decrease from 1000 µM Mg^2+^, binding exhibited the opposite effect. As the Mg^2+^ concentrations increased beyond this threshold, a reduction in receptor activation became evident (Figure 6b). This confirms reduced channel opening and, consequently, the inhibitory effects of high Mg^2+^ concentrations on P2X3 receptors. Overall, these findings underscore the complex interplay between Mg^2+^ levels and P2X3 receptor activity. While higher Mg^2+^ concentrations may dampen receptor activation, they do not necessarily abolish ligand binding, indicating a regulatory mechanism that deserves further investigation.

The heterotrimer P2X2/3 most likely consists of two subunits of P2X3 and one subunit of P2X2 [38], exhibiting a mixed response from the two receptors. Binding remains unaffected, while activation slightly decreases with an increasing Mg^2+^ concentration (Figure 6c). The heteromeric channel shows a hybrid response to Mg^2+^ coming from P2X2 and P2X3 subunits.

The modulation of Mg^2+^ on ATP-induced currents was further studied by analysing the time courses of activation and deactivation (Figure 7). The human P2X2 receptors reacted in a highly sensitive manner to small amounts of Mg^2+^ forming a complex with ATP. Even though the current response was completely abolished at 1 mM MgCl_2_ (27.5 nM ATP^4−^ + 272.5 nM Mg^2+^ATP), the time course of activation and deactivation did not show any relevant changes (Figure 7a). The human P2X3 and P2X2/3 receptors exhibited similar responses (Figure 6b,c). Mg^2+^ appeared to have little effect on activation and binding. However, any observed reduction in the current response was accompanied by changes in kinetics. For the P2X3 receptors, there was a notable slowing in the time course of desensitisation and activation, although this effect was less pronounced for *t_on_*. The estimated time constant of activation is also illustrated by a bar graph. Significant changes in the human P2X3 and P2X2/3 receptors only became evident when 97% of the total ATP (3 mM MgCl_2_ + 300 nM ATP resulted in 290.23 nM Mg^2+^ATP) was complexed with Mg^2+^. In the P2X2/3 receptors, this ATP Mg^2+^ complexation had a particularly strong impact on the time course of activation.

## 4. Discussion

ATP and their analogues can adopt different binding poses within a binding site [39,40], resulting in different ligand potencies and efficacies. These distinct pharmacological and biophysical characteristics allow for subtype-specific discrimination [1,12,15,41,42,43,44]. The agonist binding site of P2X receptors is highly conserved and does not significantly influence agonist sensitivity or efficacy. Instead, these properties are likely governed by a complex interaction involving the extracellular loop, the transmembrane, and the intracellular regions [7,8,45,46]. With the four ligands tested in this study, we showed a specific pattern for homomeric P2X2 and P2X3 receptors, as well as for the P2X2/3 heteromer.

The efficacy of the studied ligands showed big differences among the studied receptors. We observed that α,βMe-ATP activated human P2X2 receptors only partially, confirming previous results [37]. However, α,βMe-fATP exhibited a high affinity binding to P2X2 receptors, but failed to open the channel. When co-applied with ATP, there was an additive channel opening. Previous studies observed a similar effect with other nucleotides [47]. It has been demonstrated that UTP, CTP, and ADP alone do not activate P2X2 receptors [47]. However, when applied concomitantly with ATP, these ligands generated increased current amplitudes compared to ATP alone. These findings suggest that the occupancy of one ATP binding site leads to a conformational state with a change in the selectivity of the two remaining binding sites.

The ortholog binding signal coming from the fluorescent ATP derivatives allowed for relating ligand binding to activation gating in a subtype-specific manner. The binding of fATP to P2X2 receptors saturated before the activation, suggesting the existence of closed states with higher affinities. At least two potential mechanisms may account for this phenomenon: (1) P2X2 receptors have a slow desensitisation rate which accelerates at higher ligand concentrations, and their increased binding might be correlated to the degree of desensitisation. This idea is supported by previous studies on P2X1 and P2X3 receptors, where the desensitised state was attributed to a higher affinity [9,10,28,29], a phenomenon also discussed for other ligand-gated receptor proteins [48,49]. (2) The steep concentration–binding relationship might be the result of entering a “flipped” state. A previous conformational state with significant cooperative binding would occur while the channel is still closed [50,51,52].

The P2X3 receptors desensitised rapidly at all ATP concentrations, indicating a strong inherent propensity for desensitisation. Our results showed robust binding in the closed state without channel activation, suggesting a substantial disparity between activation and binding. In a living cell, the fast desensitisation of P2X3 might serve to quickly terminate signalling, preventing overstimulation, while the slower ATP-concentration-dependent desensitisation of P2X2 might allow for prolonged signalling at varying ATP levels. Generally, the data obtained herein for P2X2 and P2X3 receptors reflect their unique desensitisation kinetics and binding characteristics. While both receptor types may share the process of desensitisation upon ATP binding, the rate and ATP dependence of this process differ, leading to the observed specific responses.

When normalising for hP2X2/3 the concentration–activation and concentration–binding relationship for fATP (Figure 4c), the two curves intersect at approximately 0.5 µM, indicating that full activation occurred before complete binding. In other words, at low concentrations, fATP bound to P2X2/3 receptors without eliciting significant openings, suggesting that a single bound ligand was insufficient in activating the receptor. Moreover, the ligand acted as a partial agonist (Figure 3), exhibiting a high binding affinity while only partially activating the receptor. α,βMe-ATP showed a low potency at the P2X2/3 receptors, reflecting properties from the P2X2 and P2X3 subunits with a higher efficacy than P2X2 receptors, but an overall reduced potency. This might have been due to a binding site from a P2X3 subunit in the heteromeric P2X2/3 receptor [37].

Different metal-binding sites have been identified [11,16,17,25,53]. One metal-binding site (MBS1) predominantly binds to zinc ions, but may also have the capability to bind Mg^2+^ ions when present in high concentrations. The second metal-binding site (MBS2) mostly favours Mg^2+^ binding connected to ATP. This binding site was identified in the head domain near the binding pocket in Gulf Coast P2X [17], human P2X3 [11], and zebrafish P2X4 [53]. An acidic chamber plays an important role in this mechanism in human P2X3 receptors. Four amino acids (E109, E111, E156, and D158) have been identified that play critical roles in the binding and activation of Mg^2+^ATP. D158 and E156 are involved in stabilising ATP. E109, E111, and E156 are crucial for interacting with the ion [11,54]. It has been proposed that the adenine base of ATP interacts with one subunit and its phosphate interacts with the acidic residues of a neighbouring subunit via the Mg^2+^ ion [53]. The receptors utilise the acidic chamber to accommodate ions in two distinct modes: the ‘upper mode’ and ‘lower mode’. D158 is involved in both modes by moving the side chain towards E109 (upper mode) or γ-phosphates of ATP (lower mode). In the upper mode, Mg^2+^ is bound as a free ion. The ion may move spontaneously from the lower to the upper mode when ATP dissociates, indicating a movement pathway for divalent ions between the two modes and suggesting that ion binding in the upper mode is favoured in the absence of ATP [11].

As Mg^2+^ concentrations increased beyond 100 µM, a reduction in receptor activation became evident. Unlike activation, binding affinity did not decrease consistently with higher Mg^2+^ATP concentrations at human P2X3, and instead showed some optimisation at very high Mg^2+^ concentrations (1 mM and 3 mM). The ionic strength is likely to affect the electrostatic interactions between charged groups and Mg^2+^ ions. With an increased ionic strength, electrostatic interactions are bolstered, potentially resulting in a heightened binding affinity of Mg^2+^ to the receptor. Mg^2+^ binding to the lower mode stabilised ATP on the receptor and slowed recovery from desensitisation. By stabilising the desensitising state, fATP remained more tightly bound to the receptor, increasing Mg^2+^ binding to the receptor but drastically reducing channel opening. The substantial inhibition of P2X3 receptor activation observed at high Mg^2+^ concentrations suggests that Mg^2+^ functions as an inhibitor. From the TM pore residue, L351 is anti-correlated with the remainder of the subunit in zfP2X4 receptors [53]. We suggest that there is a modulation by Mg^2+^ through an allosteric site, inducing conformational changes by tightening the binding jaw and stabilising the ATP to the receptor in an intermediate functional state. This could explain both the increased binding affinity and the significant reduction in activation. This stabilisation led to slower transitions to the active and desensitised states, as observed. The deceleration of the activation time constant at high Mg^2+^ concentrations suggests that Mg^2+^ hampered the transition to the active state of the receptor. Overall, Mg^2+^ binding may have locked the receptor in an intermediate state, preventing full activation and elucidating the substantial slowing of the kinetic processes.

Upon comparing the sequence with the human P2X2 receptor, it becomes evident that E109 and E156 are highly conserved, unlike D158. Based on this observation, it can be inferred that D158 significantly influences Mg^2+^ATP gating and the corresponding G158 in human P2X2 hampers activation. Further, E111 is not highly conserved among the P2X receptor subtypes, and mutating glutamic acid to cysteine results in a 2-fold decrease in its potency [29]. Taking into account that the human P2X2 receptor also has a neutral amino acid (isoleucine), this might explain the right shift of the concentration–activation relationship in the presence of Mg^2+^ ions.

Heteromeric P2X2/3 receptors consist of most likely two P2X3 subunits and one P2X2 subunit, showing similar pharmacological characteristics as homomeric P2X3 receptors [38]. Their biophysical properties are dominated by the P2X2 subunit. Since each binding site has a contribution from P2X3, robust activation by Mg^2+^ATP is not surprising [38,55]. Our data show a reduced current response at high Mg^2+^ concentrations, but indicate that P2X2/3 receptors can accommodate Mg^2+^ATP complexes without a significant loss in binding affinity. The P2X3 receptors exhibited a reduced current response at high Mg^2+^ levels, while P2X2 showed no response at 1 mM Mg^2+^. Therefore, the combined effect, characterised by unaltered binding in the presence of Mg^2+^ ions, is influenced by the actions of the P2X2 and P2X3 subunits. Two possibilities may justify these observations: 1. The mechanism includes an additional low-affinity Mg^2+^ binding site. 2. An open channel block might occur, clogging the pore and leading to a decreased current response at high Mg^2+^ concentrations [56]. Further, Mg^2+^ (f)ATP binding is eventually located at the binding site between the P2X3 subunits, stabilising the binding and, thus, delaying deactivation. As a high Mg^2+^ level of 1 mM eliminates the ATP response in human P2X2 receptors, we speculate that at least one of the two binding sites is affected by a P2X2 subunit. This results in a reduced activation process that might explain both the slower activation speed and reduced amplitude at higher Mg^2+^ concentrations.

## 5. Conclusions

P2X2, P2X3, and P2X2/3 receptors have different sensitivities to ATP analogues, with specific preferences for certain ligands that distinguish their functional roles under physiological conditions. By using fluorescent ATP derivatives, we identified the subtype-specific relationships of binding and gating. Mg^2+^ affects P2X receptor activity by forming Mg^2+^ATP complexes that reduce the availability of free ATP, resulting in different effects on activation and binding across all three P2X subtypes. Exhibiting distinct binding and activation responses to Mg^2+^ATP allows for differentiating the underlying mechanisms. The interaction between ATP and Mg^2+^ significantly affects P2X receptor activity. Therefore, the central chamber region in the extracellular domain may contain multiple cation binding sites in P2X receptors.

## Figures and Tables

**Table 1:** Effects of ligands on human P2X receptors. n.d. = not determined.

Receptor	Ligand	EC_50_ [µM]	Hill	Effect (Fold in ATP Potency)	Effect (Fold in ATP Efficacy)
hP2X2	ATP	0.7 ± 0.1	1.1 ± 0.1	1	1
	α,βMe-ATP	6.8 ± 4.6	1.7 ± 1.5	0.1	0.1
	fATP	4.6 ± 0.3	1.4 ± 0.1	0.2	1.0
	α,βMe-fATP	n.d.	n.d.	n.d.	n.d.
hP2X3	ATP	1.1 ± 0.1	1.3 ± 0.2	1	1
	α,βMe-ATP	9.6 ± 3.3	1.6 ± 0.7	0.1	1.9
	fATP	1.3 ± 0.1	1.6 ± 0.1	0.9	0.8
	α,βMe-fATP	8.1 ± 0.0	3.5 ± 0.0	0.1	1.0
hP2X2/3	ATP	2.7 ± 0.4	0.9 ± 0.1	1	1
	α,βMe-ATP	n.d.	n.d.	n.d.	n.d.
	fATP	0.7 ± 0.3	0.9 ± 0.2	4.0	0.4
	α,βMe-fATP	1.5 ± 0.6	4.4 ± 3.5	1.8	0.03

## Data Availability

Data, plasmids, and software codes are available upon reasonable request.
